# Rural-urban disparities in postpartum contraceptive use among women in Nigeria: a Blinder-Oaxaca decomposition analysis

**DOI:** 10.1186/s12939-022-01674-9

**Published:** 2022-05-17

**Authors:** Obinna Princewill Anyatonwu, Miguel San Sebastián

**Affiliations:** Department of Epidemiology and Global Health, Umeå University, SE -901 87 Umeå, Sweden

**Keywords:** Contraceptive, Postpartum, Blinder-Oaxaca decomposition, Inequality, Nigeria

## Abstract

**Background:**

Unintended pregnancies are a global public health concern that could be prevented with appropriate access to contraceptive methods. Evidence from research has indicated that avoidance of closely space birth/pregnancy within the first year of postpartum, mitigates the risk of adverse health outcomes such as preterm birth, low birth-weight, etc. Postpartum family planning helps women to minimize closely spaced and unplanned pregnancies within the first 12 months after delivery. Less contraceptive use is often present in more socially disadvantaged groups. Studies from Nigeria have shown a persistent disparity on contraceptive use between rural and urban residents. To identify the factors explaining these inequalities is important to implement targeted interventions. This study aimed to identify the factors contributing to the rural-urban disparity in postpartum contraceptive use among women in Nigeria.

**Methods:**

This is a cross-sectional study using the Nigerian Demographic Health Survey. In total, 28,041 postpartum Nigerian women were included. Self-reported contraceptive use was the outcome, while the selected explanatory variables were grouped according to three theoretical perspectives: materialistic, behavioural/cultural, and psychosocial variables. Descriptive statistics and Blinder-Oaxaca decomposition were used to summarize and identify the factors contributing to the rural-urban disparity in postpartum contraceptive use.

**Results:**

In this study, 27% of women reported to have used contraceptives during the postpartum period. The rural-urban disparity in postpartum contraceptive use accounted for 18.2 percentage points. The findings further showed that the disparities in postpartum contraceptive use between rural-urban residence were mostly explained by materialistic variables (82%), followed by the behavioural/cultural variables and age (included as covariate) accounting for 15.6 and 3.0%, respectively. Household wealth (37%) and educational attainment (38%) had the most significant contribution to the differences in postpartum contraceptive use. Only 15% of the difference in postpartum contraceptive use remained unexplained.

**Conclusion:**

This study has shown important inequalities in postpartum contraceptive use between rural and urban residents in Nigeria. These differences were mainly explained by materialistic factors. These findings highlight crucial areas for the government to target in order to close the existing gap between rural and urban settings in contraceptive use in the country.

**Supplementary Information:**

The online version contains supplementary material available at 10.1186/s12939-022-01674-9.

## Background

Unintended pregnancies are a global public health issue placing mothers and babies at increased risk of morbidity and mortality [1]. In 2015–2019, it was estimated that, on average, 121 million unintended pregnancies occurred globally [2]. Studies in low- and middle-income counties (LMICs) have found that 60% of women who wanted to delay or avoid pregnancy within the first 12 months after childbirth were not using contraceptives [3, 4]. Postpartum family planning primarily focuses on avoiding closely spaced and unintended pregnancies up to 12 months post-delivery [5]. Evidence from research has indicated that avoidance of closely space birth/pregnancy within the first year of postpartum, mitigates the risk of adverse health outcomes such as preterm birth, low birth-weight, small for gestational age, premature membrane rapture, neonatal and maternal death [6, 7]. A United Nations report on the cost and benefits of contraceptive services estimates that the adoption of family planning methods would avert 1.1 million neonatal, 0.7 million infant, and 118,000 maternal deaths globally [8]. As a result, it is critical that postpartum family planning interventions identify a spectrum of contact point within the health care system that can deliver high-quality postpartum family planning services to women especially the first year following childbirth. Rustein (2008) in his study, pointed out that the risk of infant death is greatest when the time between birth and pregnancy is relatively short (12 months). However, if the time to conception is increased to 24 months and 36 months, then the risk of infant mortality would decrease considerably [9]. In the context of this study, postpartum period refers to the hours (usually 4–6 hours) following birth up to twelve-month post-delivery.

Different social factors may determine the use of contraceptives during postpartum. A recent systematic review on the factors that affect the unmet need for modern family planning methods among postpartum women in Sub-Saharan Africa included a series of aspects operating at the individual, household, and health facility levels [10]. The main factors that were significantly associated with postpartum contraceptive use at these levels included higher education, older age, parity, resumption of sexual activity, higher wealth status, prior knowledge of contraceptives, higher educational status of the husband, and his approval. Also, the use of antenatal and postnatal care services were strong determinants of postpartum contraceptive use [10]. Related studies in Nigeria have suggested inequities in family planning use due to contextual differences in family planning messages, socioeconomic and demographic characteristics, and communal composition characteristics [11]. Another comparative study on contraceptive use and its socioeconomic determinants in the country showed a persistent rural-urban disparity in the use of modern contraceptives over the years (1999, 2003, 2008, 2013), with uptake being higher in urban residents [12]. A similar pattern was found in the 2018 Nigerian Demographic Health Survey where 26% of women that lived in the urban area used contraceptives compared to 10% in the rural areas [13]. Rural women are at risk for contraceptive use disparity due to cultural disapproval, religion beliefs, partner disapproval, desire for more children, and ignorance [14, 15]. Also, distance to primary health care facilities, high cost of transportation due to poor roads, scarcity of family planning commodities and procurement of the required consumables by women were identified as problems [16, 17]. Moreover, urban women experience providers-imposed barriers to contraceptive use (in terms of age, parity, demographic characteristics), and this restriction varies across methods [18]. It is crucial to understand why these disparities in postpartum contraceptive use between rural and urban exist, by identifying the factors that explain such differences using the explanatory pathways (materialistic, behavioral and cultural). Therefore, this study aimed to ascertain the factors contributing to the rural-urban disparity in postpartum contraceptive use among women in Nigeria. The findings would not only contribute to the growing body of knowledge, but could also facilitate pragmatic response via policy formulation and be used as a tool to champion effective postpartum family planning programs. Policymakers would be able to know where to deploy limited resources in other to reduce the inequality in postpartum contraceptive use between rural and urban residence.

## Methods

### Study design and data collection

This is a secondary analysis of cross-sectional data collected through the Nigerian Demographic Health Survey (NDHS) between August and September 2018. This latest survey used computer-assisted personal interviews to collect information from respondents. A two-stage cluster sampling was undertaken. In the first stage, 1400 enumeration areas (EAs) were selected with a probability proportional to the EA size. In the second one, an equal probability systematic sampling was applied to 30 households selected from clusters identified in the previous stage. This process characterized 42,000 households. Questionnaires were translated into the three major languages (Igbo, Hausa, and Yoruba) and administered by trained field workers. Data for this study were retrieved from the women’s questionnaires. Women within the age range of 15–49 years in each sampled household were eligible for the interview [13]. A total of 41,821 women (99% response rate) were successfully interviewed. Women who had delivered a live birth within 12 months prior to the survey were eligible for this study. This resulted in an analytical sample of 28,041 women.

### Measures

The outcome variable was self-reported contraceptive use during the postpartum period. Women were asked if they or their partners were currently doing something or using any method to delay or avoid getting pregnant. Answers were expressed in a binary format as yes or no. Place of residence was used as the exposure variable and participants were grouped as either living in the rural or urban areas, irrespective of the region in Nigeria.

Explanatory variables were included if they had a plausible link to postpartum contraceptive use based on existing literature [10], fit with our explanatory pathways and were available in the NDHS dataset. The materialistic, cultural/behavioural, and psychosocial perspectives (adapted from the conceptual framework on social determinants of health) were used to organize the explanatory variables [19]. Age was used as a covariate.

The materialistic variables included maternal education, knowledge of contraceptives, distance to healthcare facility, and household wealth. Maternal education was solicited by asking respondents the highest educational level attained and categorized as: no education, primary education, secondary education, and higher education. No education was chosen as the reference category. Women who knew about contraceptives were classified as yes, the opposite as no. The latter was made the reference category. Distance to healthcare facility was operationalized using the question “Is the distance to a health facility a huge challenge when getting medical help?” and coded yes/no. The household wealth quintiles in the NDHS were calculated via the principal component analysis method using the household assets. The variable was divided in quintiles, with the first representing the poorest household and the fifth representing the richest household. The reference category was the first quintile (poorest). Religion and ethnicity were added as part of the behavioural/cultural variables. Religion was classified into the following three groups: Christians, Islam, and no religion, using Christian as the reference category. Ethnicity consisted of the following: Fulani, Igbo, Yoruba, Hausa, Igala, Ibibio, Ijaw/Izon, Kanuri/Beriberi, Tiv, Ekoi, and others. Ekoi was assigned as the reference category. Marital status was the only included variable that was classified as psychosocial due to the relationship with the social support system and its plausible link with contraceptive use. It was grouped into three categories: never-married, married, and formerly married, with the former used as the reference category. Finally, maternal age was categorized into: 15–24 years (reference), 25–34 years, and 35–49 years.

### Data analysis

Descriptive statistical analysis was performed to summarize postpartum contraceptive use and rural-urban residence across the explanatory variables. We applied weighting to adjust for the sampling procedure and non-response in all analyses. The Blinder-Oaxaca decomposition analytical method was used to estimate and decompose the disparity between rural and urban residence [20, 21]. Although this decomposition technique was developed by the aforementioned names, Neumark (1988) generalized its use [22]. Formerly, the Blinder-Oaxaca decomposition was only used to estimate linear regression models [23] but later adapted to models with a binary outcome. The decomposition analysis produces a component that explains the difference by the selected variables and a residual part indicating the non-explained difference. In the context of this study, a two-fold decomposition was carried out. Firstly, the difference in postpartum contraceptive use between rural-urban residence was estimated. Secondly, the individual contribution of each of the variables to the explained difference was estimated from the coefficients of the pooled model. The selected variables for this study were examined for missingness as well as the pattern of missingness, but no missing values were found. The variance inflation factor (VIF) was used to test for multicollinearity (mean VIF value was 1.40).

### Ethical consideration

The survey procedure and instruments used in the NDHS have ethically been approved by the National Health Research Ethics Committee of the Federal Ministry of Health of Nigeria and the Ethics Committee of the Opinion Research Corporation Macro International, Inc. (ORC Macro Inc., Calverton, MD, USA). Informed consent was sought by the trained interviewers prior to administering the questionnaire to the participants. Traces of information that could identify the study participants was made anonymous during the publication of survey findings. Permission to use and access the NDHS dataset was obtained via written consent of the DHS Program.

## Results

### Descriptive statistics

A total of 28,041 postpartum women were included in the study sample, of which 27% had ever used contraceptives during postpartum. Table 1 summarizes the characteristics of the study participants. 51% of the women enrolled in this study were within the age group of 25–34 years compared to the women within the age range of 15–24 years (16%). For the materialistic variables, knowledge of contraceptives was quite common among women (94%) compared to 6% of women who had no knowledge of contraceptive use. Around 12% of women belonged to the richest households, in contrast to one-fourth (25%) that were from the poorest households. Around one in five women belonged to the middle wealth quintile. The vast majority of women living in the urban area perceived that distance to healthcare facilities was not a big challenge (71%) in contrast to their counterparts (29%). In terms of education, 55% of women were uneducated, while about 24 and 5% of women had secondary or tertiary education, respectively. Islam was practiced by 71% of the women, compared to 28% who professed Christianity or no religion at all (1%). With regards to ethnicity, 45% of women were Hausas, followed by Igbo and Yoruba (accounting for 10% respectively). Furthermore, most of the women were married or in union (97%) compared to women who were unmarried (accounting for 1%).

**Table 1:** Weighted frequencies of explanatory variables

Variables	Frequencies	Percent
**Maternal Age (yrs.)**		
15–24	4364	16%
25–34	14,418	51%
35–49	9260	33%
**Total**	**28,042**	**100%**
***Material variables***		
**Knowledge of Contraceptive**		
No	1556	6%
Yes	26,486	94%
**Total**	**28,042**	**100%**
**Wealth quintiles**		
Poorest	7038	25%
Poor	6787	24%
Average	5974	21%
Rich	4888	18%
Richest	3355	12%
**Total**	**28,042**	**100%**
**Distance to healthcare facility**		
Not a big problem	8110	29%
A big problem	19,932	71%
**Total**	**28,042**	**100%**
**Mother’s Educational Level**		
No education	15,284	55%
Primary school education	4572	16%
Secondary school Education	6726	24%
Tertiary Education	1460	5%
**Total**	**28,042**	**100%**
***Behavioral/Cultural variables***		
**Religion**		
No religion	179	1%
Christianity	7919	28%
Islam	19,933	71%
**Total**	**28,042**	**100%**
**Ethnic Groups**		
Fulani	2701	10%
Igbo	2835	10%
Yoruba	2112	8%
Hausa	12,649	45%
Igala	170	0.6%
Ibibio	275	1%
Ijaw/Izon	307	1%
Kanuri/Beriberi	877	3%
Tiv	497	2%
Ekoi	80	0,3%
Others	5538	20%
**Total**	**28,042**	**100%**
***Psychosocial variables***		
**Marital status of women**		
Never married	240	1%
Married/In Union	27,310	97%
Formerly married/In Union	491	2%
**Total**	**28,042**	**100%**

Furthermore, most of the women were married or in union (97%) compared to women who were unmarried (accounting for 1%).

Overall, 12% of the participants referred to use a postpartum contraceptive method, of which 50.1% were using a modern method and the rest a traditional or natural one (see Additional file; Table 1s).

### Blinder-Oaxaca decomposition analysis

**Fig. 1:**
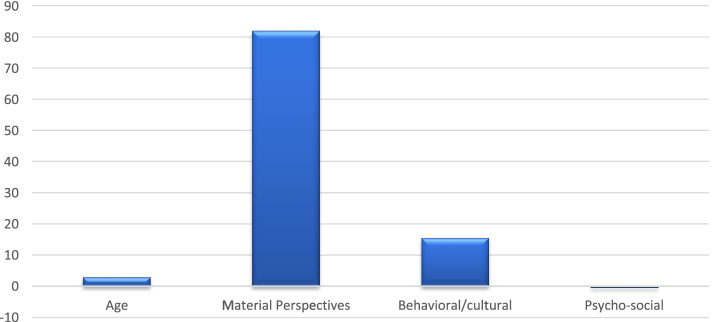
Main contributor factors (%) to the rural-urban inequality in postpartum contraceptive use

Overall, the rural-urban disparity in postpartum contraceptive use accounted for 18.2 percentage points (see Table 2). Of this aforementioned difference, 85% was explained by the selected variables, with the remaining 15% unexplained. Figure 1 depicts the materialistic, psychosocial, and behavioural/cultural aggregated variable contribution to explaining the disparity in postpartum contraceptive use between rural and urban residents. Our findings showed that the materialistic variables (82%) explained the majority of the gap existing between rural and urban residence. It is then followed by the behavioural/cultural variables, age (included as covariate), and the psychosocial variable, accounting for 15.6, 3.0%, and − 0.5%, respectively.

**Table 2:** Weighted decomposition of the disparity in postpartum contraceptive use (PPCU) between rural and urban residence in Nigeria

Overall			
Urban PPCU		40.0%	
Rural PPCU		21.8%	
Rural- Urban difference		18.2 percentage points	
Total explained difference		15.5 percentage points	
Total unexplained difference		3.0 percentage points	
Variables (***N*** = 28,041)	Coefficient	% contribution to explained difference	***P***-value
***Covariate***			
Maternal age (yrs.)			
15–24	Ref	-	-
25–34	0.002	1.1%	0.012
35–49	0.004	2.2%	< 0.001
***Material variables***			
Wealth quintiles			
Poorest	Ref	**-**	-
Poor	-0.002	-1.1%	0.144
Average	-0.000	-0.8%	0.413
Rich	0.029	15.9%	< 0.001
Richest	0.042	23.0%	< 0.001
Distance to healthcare facility			
A big problem	Ref	-	-
Not a big problem	0.006	3.3%	< 0.001
Knowledge of contraceptive			
No	Ref	-	-
Yes	0.007	3.8%	< 0.001
Mother’s educational Level			
No education	Ref	-	**-**
Primary education	0.004	2.2%	< 0.001
Secondary education	0.041	22.4%	< 0.001
Tertiary education	0.024	13.1%	< 0.001
***Behavioural/Cultural variables***			
Religion			
Christian	Ref	-	-
Islam	0.018	9.8%	< 0.001
No religion	0.001	0.5%	< 0.001
Ethnicity			
Ekoi	Ref	-	-
Fulani	-0,000	0,1%	0,956
Hausa	0,003	1,6%	0,716
Ibibio	-0,001	-0,5%	0,180
Igala	-9.47e	-0,1%	0,904
Igbo	0,003	1,6%	0,718
Ijaw/Izon	-0,001	-0,5%	0,049
Kanuri/Beriberi	- 0,002	-1,1%	0,140
Tiv	0,001	0,5%	0.376
Yoruba	0,009	5,0%	0.310
Others	-0,002	-1,1%	0.620
***Psychosocial variable***			
Marital status of women			
Never married	Ref	**-**	-
Married/In union	**-**0.005	-2.7%	< 0.001
Formerly married/In union	0.004	2.2%	< 0.001

Maternal education (38%) significantly contributed to explaining the gap in postpartum contraceptive use between rural and urban residents. Women with secondary school (22.6%) and tertiary education (13.1%) were the main categories explaining the inequality. The second important contributor, accounting for about 37% of the difference in rural-urban residence in postpartum contraceptive use, was household wealth. This finding indicates that household wealth was more concentrated among the urban residents compared to the rural residents. The cultural/behavioural variables included in this study were responsible for a smaller contribution, with Islamic religion as the most important factor (9.8%) of the inequality. Age (3.3%), distance to healthcare facilities (3.3%), and knowledge of contraceptives (3.8%) also made smaller contributions to the urban-rural gap. Ethnicity did not significantly contribute to explaining the rural-urban difference during postpartum.

## Discussion

This study used the Blinder-Oaxaca decomposition method to explain the disparity in postpartum contraceptive use between rural and urban residence in Nigeria. The findings have shown that a substantial gap (18.2 percentage points) in postpartum contraceptive use between rural and urban residence exist, favouring the urban residents. The materialistic, cultural/behavioural, and psychosocial factors explained 85% of the rural and urban contraceptive difference. Among these, maternal education, wealth quintiles, and religion accounted for the most.

The materialistic perspective variables contributed most to explaining the gap in postpartum contraceptive use among women living in rural and urban areas in Nigeria. Women from rich households were more likely to adopt contraceptives during postpartum compared to their counterparts from the rural area. A possible explanation may be that household wealth has a bearing on how a family allocates and spend their resources, healthcare included [24]. Albeit family planning is free in government health facilities, management of complications due to its use are not necessarily free coupled with ancillary fees [25]. Also, in Nigeria, the most common contraceptives (condoms and pills [13]) are often procured from medicine vendors or pharmaceutical stores. This would be a deterrent for women in the poorest households as they would want to maximize the limited resources of the family by purchasing food items and other basic household needs. A similar result was found by Rutaremwa et al. (2015), where women in higher wealth quintiles were substantially more likely than those in lower wealth quintiles to use postpartum contraceptive family planning [26]. Correspondingly, women who attained secondary and tertiary education were more likely to use contraceptives, and this favoured the urban dwellers. Education increases the receptivity to contraceptive awareness and encourage use of contraceptive to regulate childbearing [27] Also, it empowers women and proffer them the capability to challenge cultural conventions, beliefs and barriers that inhibit them from using contraceptives [28]. A related study on the micro effects of education on contraceptive use and fertility found that education increased the probability of using contraceptive which in turn influenced fertility both in urban and rural settlements. The research further revealed that the likelihood of using contraceptive increased as women climb the educational ladder [29]. In a systematic review, the impact of contraceptive education on contraceptive knowledge and decision making was examined. The findings demonstrated that diverse techniques/tools (e.g., written materials, audio/videotapes) used by researchers enhanced knowledge about contraceptive side effects, advantages of using contraceptives, the efficient method of use, and positively influenced attitudes towards contraceptive use [30]. Similar results were found in other studies that linked maternal education to contraceptive use [31, 32]. In contrast to our findings, Abraha et al. (2018) revealed that education was not significantly associated with postpartum contraceptive use [33]. Knowledge of contraceptives also contributed to increasing the gap in postpartum contraceptive use. Women who were urban residents and had prior knowledge of contraceptives were more likely to use contraceptives during postpartum compared to the rural residents. Similar findings have been reported in other studies [34]. Another contributor to the disparity in postpartum contraceptive use was the distance to a health care facility. Distance to healthcare facilities explained about 3.3% of the inequalities between rural and urban dwellers. This was expected because the vast majority of healthcare facilities and pharmacies are located in the urban area and proximity is not a huge issue compared to their counterparts. Thus, higher physical proximity can facilitate postpartum contraceptive use [35]. A study conducted in rural Ethiopia [35] was consistent with our findings.

The most important behavioural/cultural factor that contributed significantly to explaining the disparity in postpartum contraceptive use between rural and urban residents was religion (9.8%). Religion has been highlighted in other studies to influence contraceptive use. According to Obasohan (2015), contraceptive uptake was found to be lower among Muslim women compared to women from the Christian faith [36]. A possible explanation for this trend is that contraceptive use among Muslims might be considered more restricted compared to other religions [37]. Marital status also contributed to understanding the rural-urban discrepancy in postpartum contraception usage among Nigerian women. Married women were more likely to lower the inequality between rural and urban residents when compared to unmarried women. On the other hand, formerly married women increased the disparity in postpartum contraceptives. This implies that if all women were married or in union, the contraceptive difference between rural and urban dwellers would be reduced by 2.7%. Elsewhere, marital status has been associated with contraceptive use [20]. Finally, maternal age contributed positively to the inequality in postpartum contraceptive use between rural and urban women. Women within the ages of 25–49 years residing in the urban area were more likely to use contraceptives. A related study in Nigeria has reported that women between 25 and 49 years were 1.6 times more likely to use contraceptives compared to the 15–24 years group [24].

### Methodological consideration

One of the strengths of this study is that we used the NDHS which is considered a standard survey and nationally representative since data is collected from the thirty-six states in Nigeria. The high response rate (99%) of the NDHS contributed to decrease selection bias. Added to this, the sample size was large enough to investigate our research question and yield accurate estimates. Furthermore, the variables (material, cultural/behavioural, and psychosocial) included in this study had no missing values. On the other hand, contraceptive use was self-reported and thus recall bias could be operating. Other variables, such as occupation, fear of contraceptives, and job strain, that could have contributed to explaining the gap were not included due to their absence in the NDHS. Although we are aware that certain issues like number of children, child survival, postpartum amenorrhea, sexual activity and fertility desires might have affected the use of the outcome, their role in the income inequality of contraceptive use is less clear.

## Conclusions

This study has shown significant inequalities in postpartum contraceptive use between rural and urban residents in Nigeria. These inequities could be mainly explained by the materialistic factors operating in Nigeria. These findings provide crucial areas and recommendations for the government and non-governmental organizations to target in order to improve uptake of contraceptives (especially for women who has an unmet need) and close the gap between rural and urban residences that have lingered for years. Infrastructural development and investment in rural education of women by the government through the Ministry of Education could be paramount in closing the gap in postpartum contraceptive use. Conclusively, innovative, rural-friendly, and strategic ways should be instituted via policy formulation and implementation by the government to stimulate and develop the economy of the rural setting as the role of wealth in mitigating the disparity in postpartum contraceptive use cannot be undermined.

## Supplementary Information

**Additional file 1.**

## Data Availability

The datasets used and/or analyzed during the current study are available from the corresponding author on reasonable request.

## Abbreviations

LMICs: Low- and Middle-Income Countries
DHS: Demographic Health Survey
NDHS: Nigerian Demographic Health Survey
VIF: Variance Inflation Factor
