# Supplementary material for: Rural-urban disparities in postpartum contraceptive use among women in Nigeria: a Blinder-Oaxaca decomposition analysis
Source: Int J Equity Health. 2022 May 17;21:71. doi: 10.1186/s12939-022-01674-9 (PMC9116001; doi:10.1186/s12939-022-01674-9)
Supplement: Supplementary file 1 — Additional file 1. [file 12939_2022_1674_MOESM1_ESM.docx]

**Table 1s: Frequency of Postpartum Contraceptive Methods Use**

| **Contraceptive Method Frequency Percentage** | | |
| --- | --- | --- |
| Not using Contraceptive  ***Modern method***  Pills  Injection  Intrauterine device (IUD)  Male Condom  Sterilization  Implants/Nonimplant  Emergency Contraception  Other modern methods  ***Natural/traditional method***  Periodic Abstinence  Withdrawal  Other traditional Methods  Lactational amenorrhea  Standards days methods | 24618  247  59  591  327  27  449  10  3  171  406  233  892  6 | 88%  0.9%  0.2%  2.1%  1.2%  0.1%  3.2%  0.04%  0.01%  0.6%  1.5%  0.8%  1.6%  0.02% |
